# Periodontitis promotes intestinal inflammation through gut microbiota–mediated suppression of GPR109A

**DOI:** 10.3389/fcimb.2026.1761932

**Published:** 2026-02-24

**Authors:** Xinyue Wang, Zhonghan Xu, Yujie Yao, Hui Jia, Meng Du, Shuangzheng Wang, Fuhua Yan, Lili Li

**Affiliations:** 1 Nanjing Stomatological Hospital, Affiliated Hospital of Medical School, Institute of Stomatology, Nanjing University, Nanjing, China; 2 School of Medicine, Nankai University, Tianjin, China

**Keywords:** GPR109A, gut microbiota, intestinal barrier, intestinal inflammation, oral-gut axis, periodontitis

## Abstract

**Objective:**

To determine whether periodontitis promotes intestinal inflammation through gut microbiota–mediated suppression of the GPR109A receptor.

**Methods:**

Periodontitis was induced by ligatures in C57BL/6J mice under normal chow or high-fructose diet. Periodontal destruction was evaluated by micro-computed tomography and hematoxylin and eosin staining. Colonic GPR109A expression, intestinal epithelial integrity, as well as intestinal and systemic inflammation were assessed by histology and immunostaining, quantitative polymerase chain reaction (qPCR) and enzyme-linked immunosorbent assay (ELISA). Short-chain fatty acids (SCFAs) in colonic contents were quantified by GC–MS/MS. Further, the probiotic strain CBM588 was supplemented to two groups of mice (CP/LP group) to alleviate periodontitis-induced inflammation, and GPR109A expression was detected. To investigate the role of periodontitis-associated gut microbiota, fecal microbiota from control (GF-CON) and ligatured (GF-LIG) mice were transplanted into germ-free recipients, and colonic GPR109A levels and inflammatory responses were analyzed. Finally, GPR109A function was modulated by administration of GSK256073 and mepenzolate bromide in ligatured mice, and corresponding changes in tight junctional integrity as well as intestinal and systemic inflammation were evaluated.

**Results:**

Periodontitis significantly downregulated the expression of colonic GPR109A and disrupted the localization of ZO-1 and Occludin. Probiotic supplementation restored GPR109A expression and rescued ZO-1 distribution. Fecal microbiota transplantation from periodontitis donors led to GPR109A suppression, tight junction impairment, and inflammatory upregulation in germ-free mice, confirming a microbiota-dependent mechanism. Activation of GPR109A reversed barrier disruption and reduced pro-inflammatory cytokine levels.

**Conclusion:**

Periodontitis promotes colonic inflammation by gut microbiota-induced suppression of GPR109A receptor, leading to the destruction of the epithelial barrier. Activation of GPR109A restores barrier function and attenuates inflammation.

## 1 Introduction

Periodontitis is one of the most prevalent chronic inflammatory diseases worldwide, affecting nearly 50% of adults, with about 10% suffering from its severe form (Janakiram and Dye, 2020). Beyond its well-recognized role in tooth loss and impaired oral function, periodontitis has been increasingly associated with systemic disorders, including cardiovascular disease, diabetes, and inflammatory bowel disease (Kim and Pang, 2025; Hajishengallis and Chavakis, 2021). The emerging concept of the “oral-gut axis” suggests that oral dysbiosis and periodontal inflammation may influence gut homeostasis, but the precise molecular mediators remain poorly understood (Li et al., 2021; Qian et al., 2022; Bao et al., 2022).

Recent studies have highlighted the gut microbiota as a key link between oral inflammation and intestinal pathology (Xi et al., 2024). Periodontitis-associated microbial dysbiosis can extend beyond the oral cavity, altering gut microbial composition and metabolites, thereby modulating host immune responses (Bao et al., 2022; Qian et al., 2023). However, the downstream molecular targets through which the gut microbiota mediates these effects in the intestine remain unclear (Wang et al., 2024).

GPR109A is a member of the epithelial G-protein-coupled receptor (GPCR) family that functions as a metabolic sensor for microbial- and diet-derived metabolites, thereby linking luminal metabolic cues to host barrier regulation (Feng et al., 2022). Similar to other short-chain fatty acid (SCFA)–responsive receptors such as GPR41 and GPR43, as well as epithelial lipid-sensing GPCRs including GPR120 (Rubbino et al., 2022), GPR109A enables epithelial cells to sense commensal-derived butyrate, β-hydroxybutyrate, and dietary fermentation products (Chen et al., 2018; Thangaraju et al., 2009). Activation of GPR109A has been shown to strengthen epithelial barrier function, promote anti-inflammatory signaling, and protect against colonic inflammation (Macia et al., 2015). Conversely, reduced GPR109A activity is associated with heightened intestinal inflammation and susceptibility to colitis (Singh et al., 2014). Whether periodontitis and its associated microbiota influence GPR109A expression in the colon has not yet been investigated.

In this study, we hypothesized that periodontitis promotes intestinal inflammation through gut microbiota–mediated suppression of GPR109A. We first established a ligature-induced periodontitis model to examine colonic GPR109A expression. Next, we investigated whether probiotic supplementation could mitigate the impact of periodontitis on GPR109A. We then performed fecal microbiota transplantation from periodontitis mice to germ-free recipients to assess causality. Finally, we tested whether pharmacological activation of GPR109A could attenuate periodontitis-associated colonic inflammation. Collectively, our work uncovers a novel oral–gut axis mechanism whereby periodontitis impairs intestinal homeostasis through GPR109A suppression, suggesting new therapeutic opportunities targeting this pathway.

## 2 Materials and methods

### 2.1 Ethical approval and study design

All animal experiments followed the National Institutes of Health guidelines (NIH publication no. 85-23, revised 1996) and were approved by the Animal Ethics Committee of Nanjing University (IACUC-D2303078, China). All mice were humanely sacrificed at the end of the respective experimental period via administration of an overdose of pentobarbital sodium (200 mg/kg, Xiya) in accordance with the AVMA Guidelines on Euthanasia (American Veterinary Medical Association, 2020), ensuring complete loss of consciousness prior to death.

### 2.2 Mouse models and experimental groups

Seven-week-old male C57BL/6J mice (GemPharmatech, China) were housed under SPF conditions (3–4 per cage) and maintained on a normal chow diet or a 60% high-fructose diet (XT704, Xietong bio., China). After acclimation, 28 animals were randomly assigned to four groups: ND_CON, mice fed with a standard chow diet and without ligature placement (normal control); ND_LIG, mice fed with a standard chow diet and subjected to ligature-induced periodontitis; HFD_ CON, mice fed with a 60% high-fructose diet without ligature placement; HFD_LIG, mice fed with a 60% high-fructose diet and subjected to ligature-induced periodontitis (n = 7 per group). At week 8, fecal pellets from HFD_ CON and HFD_LIG groups were collected and preserved in saline containing glycerol (30%) and cysteine (0.1%). All animals were sacrificed at week 9.

### 2.3 Induction of periodontal inflammation

Mice were anesthetized via intraperitoneal injection of pentobarbital sodium (40 mg/kg, Xiya) combined with atropine sulfate monohydrate (0.1 mg/kg, KingYork). Then, periodontitis was induced by placing silk ligatures around maxillary second molars under anesthesia as previously described (Li et al., 2021). Ligatures were monitored every 48 h and replaced as needed to ensure continuous irritation.

### 2.4 Intervention with probiotics

To assess the effect of SCFA-producing strains, 28 mice were randomized into four groups (n = 7 each): CON, mice without ligature placement or probiotic treatment; LIG, mice with ligature-induced periodontitis, without probiotic treatment; CP, mice with probiotic administration only, without ligature placement; LP, mice with both ligature-induced periodontitis and probiotic treatment. All groups were maintained on a high-fructose diet throughout the experiment. Periodontitis was induced over 9 weeks. Probiotic CBM588 (Miyarisan, Japan) was delivered by gavage three times per week during the final 5 weeks.

### 2.5 Culture and identification of CBM588

CBM588 was prepared as reported previously (Zhang et al., 2023). Briefly, bacterial powder was dissolved in Reinforced Clostridial Medium (RCM; Hopebiol, Qingdao), incubated overnight at 37 °C, streaked onto RCM agar, and cultured for 24 h. Colonies were subcultured, and growth was monitored at OD600. Log-phase cultures (OD600 = 0.6–0.8) were centrifuged (1500 g, 15 min, 4 °C), resuspended, and adjusted to 10^9^ CFU/mL. Species identification was confirmed by 16S rRNA sequencing and NCBI BLAST alignment.

### 2.6 Germ-free colonization

Germ-free male C57BL/6J mice (n = 10, 7 weeks old; GemPharmatech, China) were maintained in sterile isolators with autoclaved food and water. They were randomized into two groups (GF-CON, GF-LIG; n = 5 each) and colonized with fecal suspensions derived from either control or periodontitis donors. Fecal samples from donor mice within each group were pooled prior to transplantation. Oral gavage was performed three times weekly for 8 weeks as described previously (Qian et al., 2023).

### 2.7 Preparation of donor microbiota

Frozen fecal samples were thawed at 37 °C and processed under anaerobic conditions. After homogenization and filtration (70-mesh), the suspension was centrifuged (4000 rpm, 15 min, 4 °C), washed, resuspended in preservation buffer, and stored at −80 °C until transplantation (Qian et al., 2023).

### 2.8 GPR109A agonist and inhibitor intervention

To investigate the role of GPR109A, 32 mice were randomized into four groups (n = 8 each): CON, mice without ligature placement, administered sterile saline; LIG, mice with ligature-induced periodontitis, administered sterile saline; LG, mice with GPR109A agonist GSK256073 administration and ligature placement; LM, mice with ligature-induced periodontitis and the GPR109A inhibitor, mepenzolate bromide treatment. All groups were maintained on a high-fructose diet throughout the experiment. Periodontitis was induced over 9 weeks. GPR109A agonist/inhibitor or sterile saline was delivered by gavage three times per week during the final 5 weeks.

### 2.9 Micro-CT analysis

After fixation, maxillae were scanned using a Skyscan 1172 system (Bruker, Belgium) at 18 μm resolution. The cemento-enamel junction–alveolar bone crest (CEJ–ABC) distance was calculated with CTAn and DataViewer software.

### 2.10 Histology and immunostaining

Decalcified maxillae (Servicebio, Wuhan, China) were paraffin-embedded, sectioned, and stained with hematoxylin and eosin (H&E) for histological assessment, and colon tissues were processed in parallel. For immunofluorescence, colonic sections were incubated with anti-ZO-1 tight junction protein antibody (ab221547, Abcam, UK) and anti-Occludin antibody (ab216327, Abcam, UK), followed by fluorescence detection and image acquisition using a PANNORAMIC MID scanner (3DHISTECH, Hungary). For immunohistochemistry, colonic sections were incubated with an anti-GPR109A antibody (A15611, ABclonal, China), visualized with a DAB detection kit (Servicebio, Wuhan, China), counterstained with hematoxylin, and scanned with the same digital slide scanner.

### 2.11 Quantitative PCR

Total RNA was extracted using TRIzol reagent (Invitrogen, USA) and quantified with a Nanodrop 2000 (Thermo Scientific). Reverse transcription was performed with PrimeScript RT Master Mix (Takara), and qPCR was carried out using SYBR^®^ Select Master Mix (Applied Biosystems). Gene expression was normalized to GAPDH and analyzed by the 2^^-^ΔΔCt^ method. Primer sequences are provided in **Supplementary Table S1**.

### 2.12 Short-chain fatty acids measurement

SCFAs in colonic contents were quantified by GC–MS/MS. Briefly, 20 mg of sample was homogenized in 1 mL 0.5% (v/v) phosphoric acid, vortexed and ultrasonicated, and centrifuged at 12,000 rpm for 10 min at 4 °C. 100 μL of the supernatant was extracted with 500 μL methyl tert-butyl ether (MTBE) containing internal standard, followed by vortexing, ultrasonication, and centrifugation under the same conditions. The organic phase was collected for analysis. GC–MS/MS analysis was performed using an Agilent 7890B gas chromatograph coupled to an Agilent 7000D triple quadrupole mass spectrometer with a DB-FFAP column (30 m × 0.25 mm i.d., 0.25 μm). Helium was used as the carrier gas at 1.2 mL/min. Samples (1 μL) were injected in split mode (5:1). The oven program was 50 °C for 1 min, increased to 220 °C at 18 °C/min, and held for 5 min. Data were acquired in MRM mode, with injector and transfer line temperatures set to 250 °C and 230 °C, respectively. SCFAs were quantified using external calibration standards prepared in MTBE (1 mg/mL) and normalized to the internal standard.

### 2.13 Statistical analysis

Statistical evaluations were carried out with GraphPad Prism version 9.5.0 (GraphPad Software, San Diego, USA). Data are presented as mean ± standard deviation (SD), with 5–8 mice per group. Normality was assessed by the Shapiro-Wilk test and homogeneity of variance by Levene’s test. For data that met parametric assumptions, differences between two groups were examined using unpaired Student’s t-test, while multiple groups were compared by one-way analysis of variance (ANOVA) followed by appropriate *post hoc* testing. When the distribution deviated from normality, non-parametric alternatives (Mann-Whitney U test) were used. Statistical significance was defined as P < 0.05.

## 3 Results

### 3.1 Periodontitis leads to decreased Colonic GPR109A

**Figure 1:**
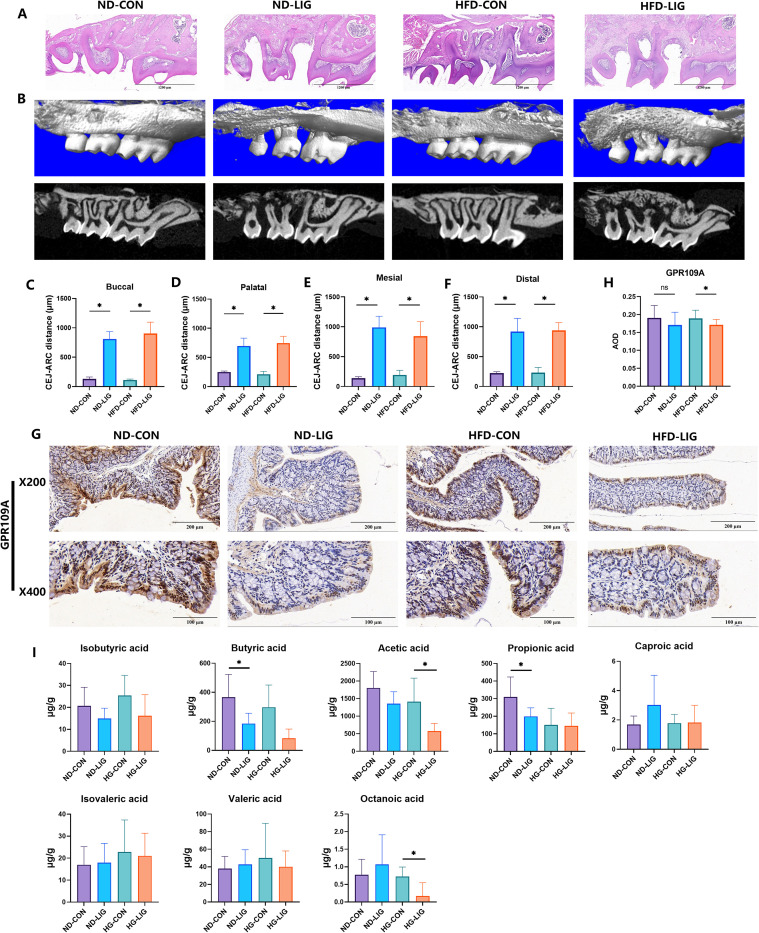
Periodontitis downregulates intestinal GPR109A expression in mice. Representative H&E-stained maxillary sections **(A)** and micro-CT slices **(B)** showing successful induction of periodontitis, with evident soft tissue destruction and alveolar bone loss compared with controls. **(C-F)** Micro-CT quantification demonstrated significant alveolar bone loss at distal, mesial, buccal, and palatal sites in the periodontitis group. **(G, H)** Representative immunohistochemistry of colonic GPR109A. Staining intensity is significantly reduced in HFD_LIG group compared with HFD_CON group. **(I)** Quantification of colonic contents revealed that acetic acid and octanoic acid levels were significantly reduced in the HFD_LIG group compared with the HFD_CON group, while butyric acid and isobutyric acid exhibited a decreasing trend. Data were expressed as mean ± SD (n = 7 animals/group). *P < 0.05. ND_CON, control; ND_LIG, periodontitis; HFD_CON, high-fructose diet control; HFD_LIG, high-fructose diet and periodontitis.

Histological examination with H&E staining and reconstructed micro-CT images confirmed successful induction of ligature-induced periodontitis, characterized by marked soft tissue destruction and alveolar bone resorption relative to controls (**Figures 1A, B**). Quantitative analysis of micro-CT parameters further demonstrated significant alveolar bone loss at buccal, palatal, mesial, and distal sites in periodontitis groups (**Figures 1C-F**).

Importantly, immunohistochemical staining of colonic tissues revealed that GPR109A expression was markedly decreased in the HFD_LIG group compared with the HFD_CON group (**Figures 1G, H**). Analysis of colonic contents revealed a general downward trend in the levels of short-chain fatty acids (SCFAs) and selected medium-chain fatty acids (**Figure 1I**). The levels of acetic acid and octanoic acid were significantly reduced in the HFD_LIG group compared with the HFD_CON group, while isobutyric acid and butyric acid exhibited a decreasing trend but did not reach statistical significance. Given that GPR109A functions as a sensor for microbial metabolites, these metabolic alterations suggest that the downregulation of colonic GPR109A is likely associated with the reduced availability of its microbial ligands.

**Figure 2:**
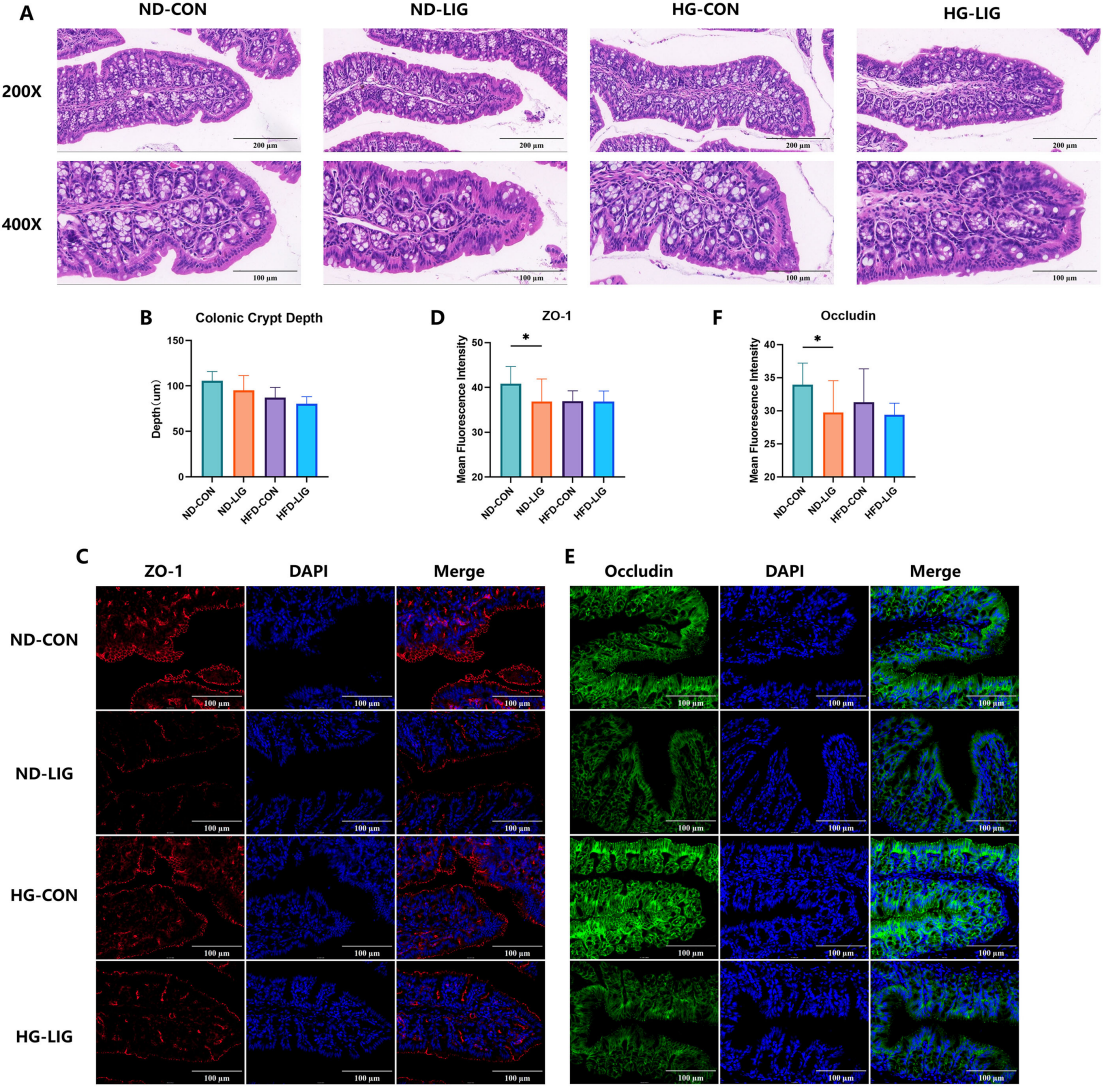
Periodontitis disrupts intestinal barrier integrity in mice. **(A)** Representative H&E-stained colonic sections showing mucosal architecture in control mice and periodontitis mice. **(B)** Quantification of crypt depth revealed no significant differences between groups. **(C, D)** Representative immunofluorescence staining of ZO-1 (red) and quantitative analysis demonstrated reduced ZO-1 expression in the periodontitis group compared with controls. **(E, F)** Representative immunofluorescence staining of Occludin (green) and quantitative analysis showed diminished Occludin localization along the epithelial barrier in periodontitis mice. Data were expressed as mean ± SD (n = 7 animals/group). *P < 0.05. ND_CON, control; ND_LIG, periodontitis; HFD_CON, high-fructose diet control; HFD_LIG, high-fructose diet and periodontitis.

To evaluate the effect of periodontitis on intestinal barrier function, histological and immunofluorescence analyses were performed on colonic tissues. H&E staining revealed no obvious differences in overall mucosal architecture between the HFD_LIG and the HFD_CON groups, as well as between the ND_LIG and ND_CON groups (**Figure 2A**). Consistently, quantification of crypt depth showed no significant alterations in either the HFD_LIG group or the ND_LIG group when compared with their respective control groups (**Figure 2B**). In contrast, immunofluorescence staining demonstrated that ZO-1 and Occludin expression were significantly reduced in the ND_LIG group compared with the ND_CON group, whereas no significant differences were observed between the HFD_LIG and HFD_CON groups (**Figures 2C-F**). These findings indicate that although gross colonic morphology remains largely intact, periodontitis compromises epithelial barrier integrity through downregulation of tight junction proteins.

### 3.2 Probiotic supplementation mitigates periodontitis-induced suppression of colonic GPR109A

**Figure 3:**
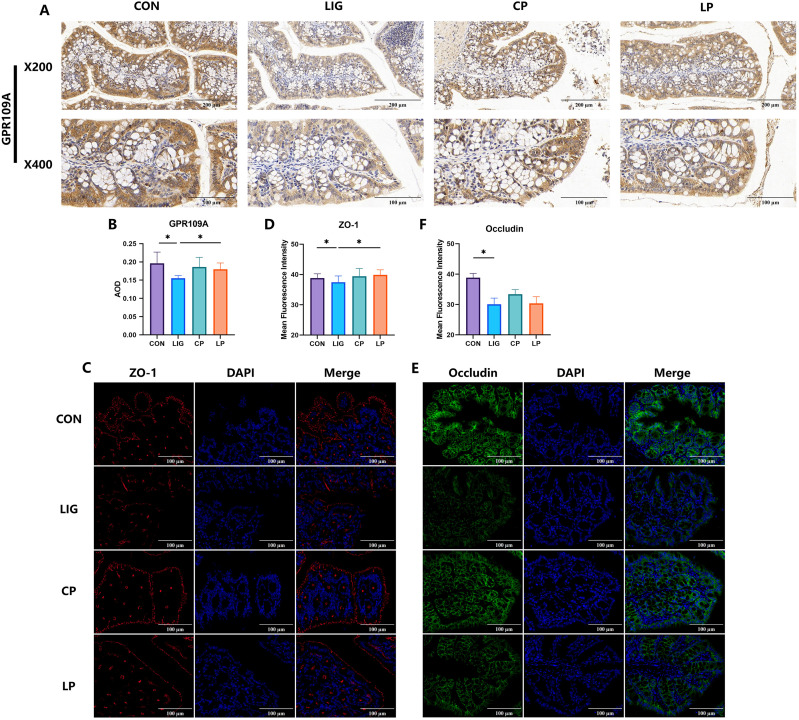
Probiotic supplementation mitigates periodontitis-induced suppression of colonic GPR109A. **(A, B)** Representative immunohistochemistry and quantitative analysis of colonic GPR109A showing decreased staining intensity in the periodontitis group compared with controls, which was restored upon probiotic supplementation. **(C, D)** Representative immunofluorescence staining of ZO-1 (red) and quantitative analysis demonstrated that ZO-1 expression was reduced in the periodontitis group but was restored after probiotic supplementation. **(E, F)** Representative immunofluorescence staining of Occludin (green) and quantitative analysis showed diminished Occludin localization along the epithelial barrier in periodontitis mice, with no significant improvement following probiotic treatment. Data were expressed as mean ± SD (n = 7 animals/group). *P < 0.05. CON, control; LIG, periodontitis; CP, probiotic supplementation; LP, periodontitis with probiotic supplementation.

To determine whether correction of periodontitis-associated gut microbiota dysbiosis by probiotic supplementation could relieve the suppression of colonic GPR109A, we carried out immunohistochemistry analyses. Colonic GPR109A expression was significantly reduced in the periodontitis group compared with controls, while probiotic supplementation restored its staining intensity (**Figures 3A, B**).

In addition to GPR109A, we assessed the expression of tight junction proteins. ZO-1 immunofluorescence showed markedly decreased expression in periodontitis mice, which was restored by probiotic supplementation (**Figures 3C, D**). For Occludin, while its localization was diminished in the periodontitis group compared with controls, probiotic treatment did not lead to a significant improvement(**Figures 3E, F**). These results demonstrate that probiotic supplementation mitigates periodontitis-induced suppression of colonic GPR109A and restores ZO-1-mediated tight junction integrity.

### 3.3 Fecal microbiota from periodontitis mice transmits colonic GPR109A suppression to germ-free mice

16S rRNA sequencing of fecal samples from HFD_CON and HFD_LIG mice confirmed significant restructuring of the gut microbiota, marked by reduced alpha diversity and distinct community clustering. Moreover, specific alterations were observed in taxa associated with SCFA metabolism, characterized by reduced *Alistipes*, *Blautia*, *Butyricimonas*, *Colidextribacter*, *Rikenella*, and *Anaerotruncus_colihominis*, alongside enriched *Odoribacter*, *Clostridia_UCG-014*, *Caproiciproducens*, and members of the Erysipelotrichaceae and RF39 lineages (**Supplementary Figure 1**).

**Figure 4:**
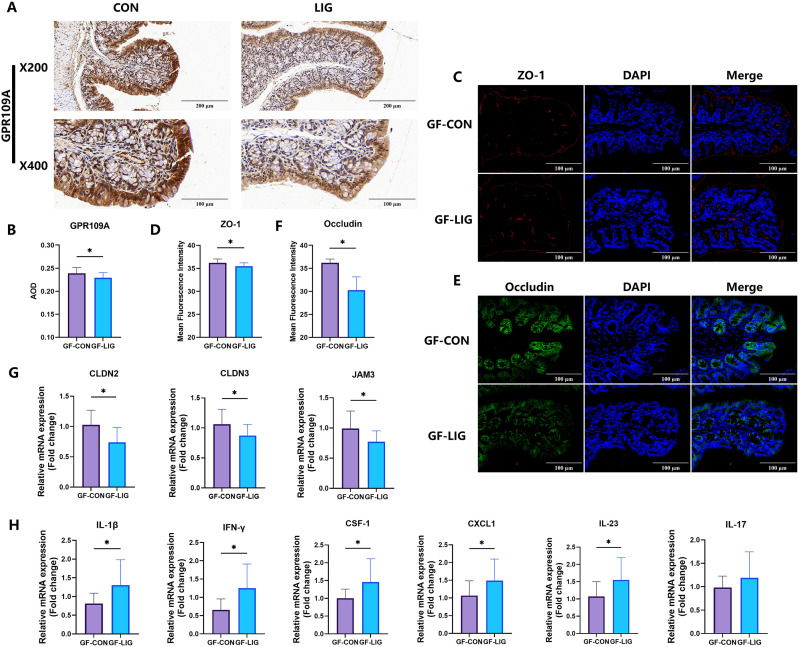
Fecal microbiota from periodontitis mice transmits colonic GPR109A suppression to germ-free mice. **(A, B)** Representative immunohistochemistry and quantitative analysis of colonic GPR109A showing decreased staining intensity in germ-free mice receiving fecal microbiota from periodontitis donors (GF-LIG) compared with germ-free controls (GF-CON). **(C, D)** Representative immunofluorescence staining of ZO-1 and quantitative analysis demonstrated reduced localization in GF-LIG mice. **(E, F)** Representative immunofluorescence staining of Occludin and quantitative analysis showed diminished epithelial localization in GF-LIG mice compared with controls. **(G)** qPCR analysis of tight junction proteins (CLDN2, CLDN3, and JAM3) revealed decreased expression in GF-LIG mice. **(H)** qPCR analysis of colonic inflammatory cytokines (IL-1β, IFN-γ, CSF-1, CXCL-1, and IL-23) demonstrated significant upregulation in GF-LIG mice. Data were expressed as mean ± SD (n = 5 animals/group). *P < 0.05. GF-CON, germ-free control; GF-LIG, germ-free mice receiving fecal microbiota from periodontitis donors.

To assess whether this restructured microbial community is responsible for the suppression of colonic GPR109A in periodontitis, we transplanted fecal microbiota from these donor groups into germ-free recipients. Immunohistochemistry revealed that colonic GPR109A expression was markedly reduced in GF-LIG mice compared with GF-CON, and quantitative analysis confirmed this downregulation (**Figures 4A, B**).

In addition, tight junction integrity was impaired following fecal microbiota transplantation from periodontitis donors. ZO-1 expression was diminished in GF-LIG mice as shown by immunofluorescence and quantitative analysis (**Figures 4C, D**). Similarly, Occludin localization along the epithelial barrier was reduced (**Figures 4E, F**), and qPCR analysis confirmed decreased mRNA expression of CLDN2, CLDN3, and JAM3 in GF-LIG mice relative to controls (**Figure 4G**).

Moreover, colonic inflammation was significantly exacerbated in GF-LIG mice. qPCR analysis showed elevated expression of pro-inflammatory cytokines including IL-1β, IFN-γ, CSF-1, CXCL-1, and IL-23 (**Figure 4H**). Together, these findings demonstrate that fecal microbiota from periodontitis mice transmits colonic GPR109A suppression, compromises epithelial barrier integrity, and promotes inflammatory responses in germ-free recipients.

### 3.4 Activation of GPR109A attenuates periodontitis-associated colonic inflammation

**Figure 5:**
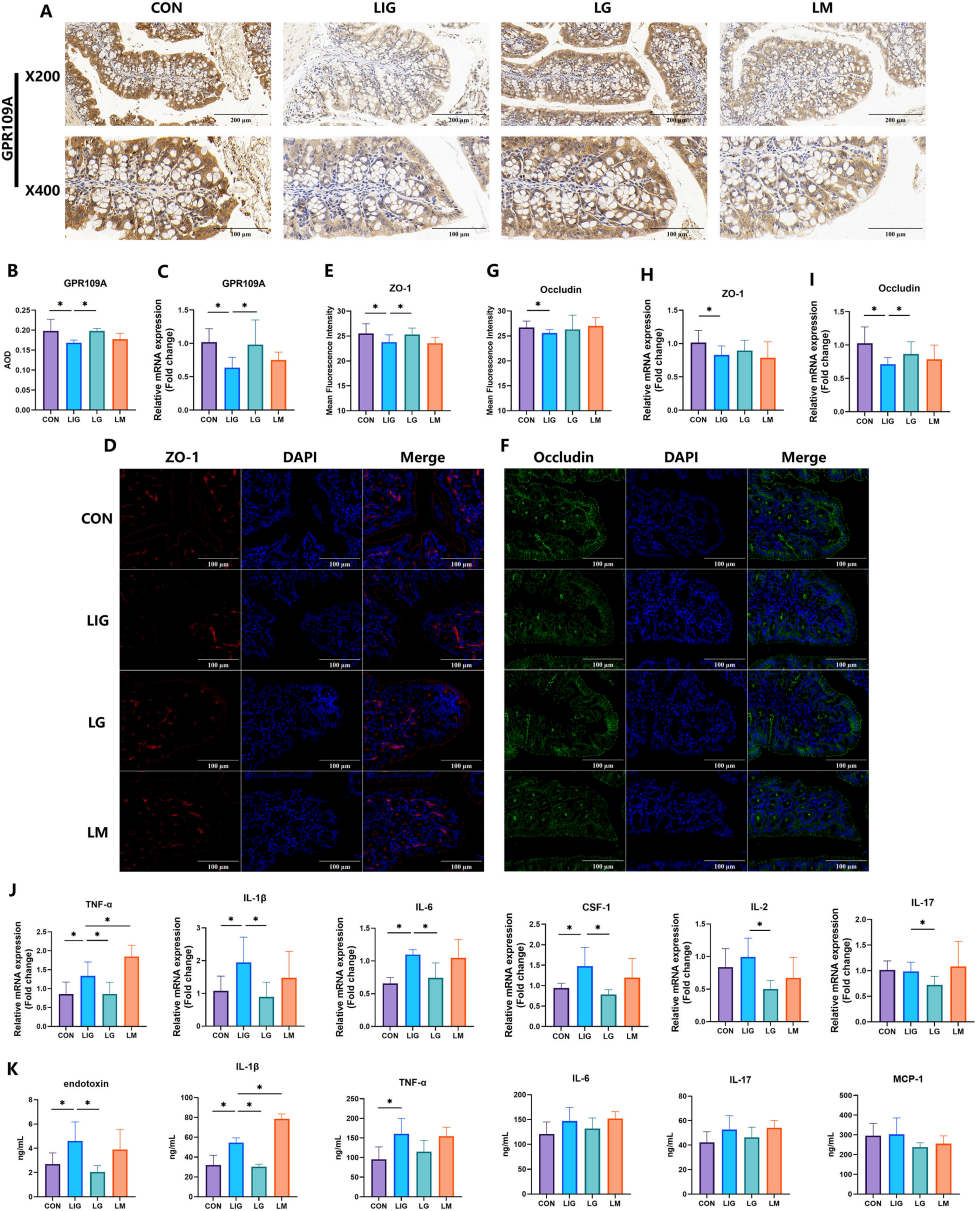
Activation of GPR109A attenuates periodontitis-associated colonic inflammation. **(A, B)** Representative immunohistochemistry and quantitative analysis of colonic GPR109A showing reduced expression in the periodontitis group (LIG). Expression was restored following treatment with the GPR109A agonist GSK256073 (LG), but was not significantly altered by the GPR109A antagonist mepenzolate bromide (LM). **(C)** qPCR analysis of colonic GPR109A mRNA expression revealed similar trends. **(D, E)** Representative immunohistochemistry of ZO-1 and quantitative analysis demonstrated decreased expression in the periodontitis group, which was improved by GPR109A activation. **(F, G)** Representative immunohistochemistry of Occludin and quantitative analysis showed diminished localization in periodontitis mice, partially restored by agonist treatment. **(H, I)** qPCR analysis of Occludin and ZO-1 revealed similar trends. **(J)** qPCR analysis of colonic inflammatory cytokines (TNF-α, IL-1β, IL-6, CSF-1) showed increased expression in periodontitis mice, attenuated by agonist treatment, whereas TNF-α expression was aggravated by antagonist treatment. **(K)** ELISA results demonstrated significantly elevated serum levels of endotoxin and IL-1β in the LIG group. Compared to the LIG group, the LG group showed significant reductions in endotoxin, IL-1β, and TNF-α, whereas the LM group exhibited a significant increase in IL-1β. Data were expressed as mean ± SD (n = 8 animals/group). *P < 0.05. CON, control; LIG, periodontitis; LG, periodontitis + GSK256073 (GPR109A agonist); LM, periodontitis + mepenzolate bromide (GPR109A antagonist).

To assess the functional role of GPR109A in periodontitis-associated intestinal pathology, we treated mice with the selective GPR109A agonist GSK256073 or the antagonist mepenzolate bromide. Colonic GPR109A expression was markedly reduced in the periodontitis group (LIG), whereas agonist treatment restored its expression, while antagonist administration did not result in a significant difference (**Figures 5A-C**).

Consistent with these findings, tight junction integrity was regulated in a GPR109A-dependent manner. ZO-1 expression was decreased in periodontitis mice and improved by GPR109A activation (**Figures 5D, E**). Similar trends were observed for Occludin, where agonist treatment partially restored its epithelial localization (**Figures 5F, G**). qPCR analysis of Occludin and ZO-1 mRNA levels confirmed these results (**Figures 5H, I**).

Importantly, modulation of GPR109A signaling also affected colonic inflammation. Periodontitis induced significant upregulation of pro-inflammatory cytokines including TNF-α, IL-1β, IL-6 and CSF-1, which were attenuated by GPR109A agonist treatment (**Figure 5J**). ELISA assays of colonic homogenates further demonstrated that levels of endotoxin and IL-1β followed the same pattern, being elevated in periodontitis mice and reduced with GPR109A activation (**Figure 5K**).

Together, these results demonstrate that GPR109A plays a protective role in maintaining intestinal barrier integrity and limiting inflammatory responses during periodontitis, and pharmacological activation of GPR109A effectively attenuates periodontitis-associated colonic inflammation.

## 4 Discussion

In this study, we provide systematic evidence that periodontitis disturbs intestinal homeostasis through gut microbiota-mediated suppression of colonic GPR109A. Using a ligature-induced periodontitis model, we confirmed typical periodontal tissue destruction and alveolar bone loss. Importantly, we demonstrated that colonic GPR109A expression was significantly reduced in periodontitis mice, concomitant with disruption of epithelial barrier integrity. Probiotic supplementation alleviated these pathological changes, while fecal microbiota transplantation from periodontitis mice transmitted the phenotype of reduced colonic GPR109A, barrier dysfunction, and enhanced inflammation to germ-free recipients. Furthermore, pharmacological modulation of GPR109A signaling revealed that receptor activation attenuated intestinal inflammation.

Our previous work demonstrated that periodontitis induces low-grade intestinal inflammation and alters gut microbial composition (Li et al., 2021), and that transplantation of dysbiotic microbiota from periodontitis mice transfers this inflammatory phenotype to recipient mice (Li et al., 2022). However, the precise mechanisms by which gut microbiota disrupts the intestinal epithelial barrier and the key molecular targets involved have remained unclear. Given that we previously observed significant alterations in butyrate-producing bacteria that played a crucial role in mediating hyperglycemia associated with periodontitis (Li et al., 2021), we hypothesized that the butyrate receptor GPR109A might serve as a critical mediator in this process. In the present study, we tested this hypothesis using complementary approaches, including probiotic supplementation to correct gut dysbiosis, fecal microbiota transplantation, and pharmacological activation of GPR109A. These findings provide direct evidence that GPR109A is a central link between periodontitis-associated gut dysbiosis, epithelial barrier dysfunction, and intestinal inflammation.

In our experimental design, we introduced a high-fructose diet to capture both clinical relevance and mechanistic plausibility. High-sugar diets are highly prevalent in modern societies and represent a widespread suboptimal lifestyle that affects large segments of the population (Ge et al., 2025; Lara-Castor et al., 2025). Such diets are well recognized to induce low-grade systemic inflammation and to destabilize the gut microbiota and intestinal epithelial barrier (Zhang et al., 2024; Satokari, 2020). Under these conditions of impaired homeostasis, periodontal inflammation is more likely to exert pronounced systemic effects, including further disruption of the gut barrier. This interaction is particularly pertinent to metabolically compromised populations, where the burden of periodontitis may be amplified (Reytor-González et al., 2024). By modeling periodontitis in the context of a high-fructose diet, our study reflects real-world dietary exposures and enhances translational relevance, especially given the strong association of high-sugar intake with metabolic syndrome, obesity, and diabetes.

The oral–gut axis has emerged as an important research focus, as accumulating evidence suggests that oral dysbiosis can influence gut microbiota composition and intestinal inflammation (Azzolino et al., 2025). Epidemiological studies have linked periodontitis with an increased risk of inflammatory bowel disease (IBD) (Park et al., 2025), metabolic disorders (Saito et al., 2024), and systemic low-grade inflammation (Cecoro et al., 2020; Pink et al., 2023). However, the molecular mediators underpinning this association remain poorly understood. Our findings extend current knowledge by demonstrating that GPR109A, a receptor for niacin and short-chain fatty acids such as butyrate, is a crucial target suppressed in the colonic mucosa of periodontitis mice.

As a key member of the epithelial metabolite-sensing GPCR family, GPR109A serves as a central regulator linking microbial and dietary metabolites to the maintenance of mucosal barrier integrity and immune balance (Chang, 2024). Activation of GPR109A by microbiota-derived butyrate or ketone bodies not only strengthens epithelial tight-junction assembly and mucus layer organization, but also modulates epithelial–immune crosstalk by promoting anti-inflammatory signaling and restraining excessive NF-κB– and inflammasome-driven responses (Gong et al., 2020; Chen et al., 2018). This places GPR109A alongside other SCFA-responsive receptors and lipid-sensing GPR120 as part of a coordinated signaling network that translates luminal metabolic activity into protective epithelial programs, including reinforcement of barrier function, regulation of goblet cell differentiation, and control of cytokine and antimicrobial peptide production (Dias Nirello et al., 2025; Takakuwa et al., 2019; Rubbino et al., 2022). Thus, GPR109A has been established as an important regulator of mucosal immunity and epithelial integrity (Liu et al., 2022; Gong et al., 2020). Previous studies have shown that GPR109A deficiency leads to increased susceptibility to colitis and colorectal carcinogenesis (Singh et al., 2014). In line with these findings, we observed that colonic GPR109A expression was markedly reduced in periodontitis, accompanied by diminished expression of the tight junction proteins ZO-1 and Occludin(**Figures 2D, F**). Notably, this epithelial barrier dysfunction precedes overt inflammatory pathology, as hematoxylin–eosin staining of colonic tissues revealed no apparent histological damage or inflammatory cell infiltration in periodontitis mice (**Figure 2A**). These findings indicate that disruption of epithelial tight junctions occurs at an early stage and is not a secondary consequence of established inflammation. Consistent with this interpretation, pharmacological activation of GPR109A significantly attenuated inflammatory responses in periodontitis mice (**Figure 5**), supporting a functional role for GPR109A signaling in maintaining epithelial barrier integrity. In this context, suppression of GPR109A may primarily compromise epithelial tight junction organization, thereby increasing mucosal permeability and creating a permissive environment for subsequent inflammatory activation.

Interestingly, we found that the GPR109A antagonist mepenzolate bromide did not further exacerbate intestinal tight junction disruption or induce a broad increase in inflammatory cytokine expression in periodontitis-challenged mice. Mepenzolate bromide is a well-characterized functional antagonist of GPR109A that primarily inhibits receptor signaling rather than directly downregulating receptor expression, which may partly explain the absence of additional suppression of GPR109A levels. Consistent with this mechanism, previous studies have shown that pharmacological inhibition of GPR109A with mepenzolate bromide effectively blocks receptor activity without reducing GPR109A protein expression (Yang et al., 2024). Moreover, because periodontitis itself markedly downregulates colonic GPR109A expression, GPR109A-mediated protective signaling is likely already substantially compromised, thereby limiting the residual receptor pool available for further pharmacological antagonism. This interpretation is supported by prior studies demonstrating that, in disease contexts characterized by reduced GPR109A expression or function, genetic deletion or pharmacological antagonism of GPR109A does not further worsen pathological indices, but instead primarily abolishes the protective effects conferred by receptor activation (Snelson et al., 2020; Kang et al., 2022).

The fecal microbiota transplantation experiments provide compelling evidence that gut microbiota is responsible for mediating the suppression of GPR109A. To further elucidate the microbial basis underlying the functional alterations observed after fecal microbiota transplantation, we performed 16S rRNA gene sequencing of donor fecal samples. Our results showed that periodontitis was associated with a pronounced restructuring of the gut microbial community, characterized by reduced microbial diversity, distinct compositional alterations, and significant shifts in multiple taxa implicated in short-chain fatty acid metabolism. Interestingly, Germ-free mice colonized with fecal microbiota from periodontitis donors recapitulated the phenotype of reduced GPR109A expression, impaired barrier function, and elevated pro-inflammatory cytokines. These findings highlight the causal role of dysbiotic microbiota in transmitting the pathological effects of periodontitis to the intestine. The decreased activation of this receptor subsequently compromises barrier integrity and enhances inflammation (Chen et al., 2018; Gong et al., 2020). In addition, dysbiotic microbiota may directly promote the expansion of pro-inflammatory bacterial taxa and trigger host immune responses that further downregulate GPR109A expression (Maciel-Fiuza et al., 2023).

Notably, our data show that supplementation with a butyrate-producing probiotic strain effectively restored colonic GPR109A expression and normalized tight junction protein localization. By specifically enhancing microbial butyrate output, this intervention targeted GPR109A activation, thereby underscoring the central role of this receptor in mediating periodontitis-associated intestinal pathology. These results highlight the potential of butyrate-producing probiotics to counteract dysbiosis and reinforce host–microbiota metabolic crosstalk critical for GPR109A signaling (Gong et al., 2020). Previous studies have reported beneficial effects of probiotics in reducing systemic inflammation and improving gut barrier integrity (Zheng et al., 2023; Ghorbani et al., 2025), and our findings extend this evidence by linking probiotic effects specifically to GPR109A activation. This suggests that therapeutic strategies aimed at the microbiota–butyrate–GPR109A axis may not only mitigate intestinal inflammation but also attenuate the systemic consequences of periodontitis.

Our findings have important implications for both periodontal and gastrointestinal health. First, they reinforce the concept that periodontitis is not merely an oral disease but may act as a driver of distal intestinal pathology through microbial and molecular pathways. This highlights the importance of comprehensive periodontal care in reducing the risk of systemic inflammatory diseases, including IBD (Madsen et al., 2023; Ozayzan et al., 2024). Second, identifying GPR109A as a key mediator provides a tangible molecular target for intervention, suggesting that restoring its signaling, such as via agonists like GSK256073 or supplementation with probiotics, could serve as a viable strategy to manage patients with both periodontal disease and intestinal inflammatory disorders.

Despite the strengths of our study, several limitations should be acknowledged. First, our findings are based on murine models, and translation to human physiology requires careful validation. Although ligature-induced periodontitis and fecal microbiota transplantation provide robust tools to study oral–gut interactions, human studies are essential to confirm whether similar mechanisms operate in clinical populations. Second, while we demonstrated the involvement of gut microbiota in GPR109A suppression, we did not identify specific bacterial taxa or metabolites responsible. Future work employing metagenomic sequencing and metabolomic profiling will be necessary to pinpoint the microbial drivers of this effect, with particular attention to butyrate-producing bacteria. Third, our study focused primarily on colonic outcomes, but systemic consequences of GPR109A modulation, including effects on cardiovascular or metabolic health, remain unexplored. Finally, although we used a selective agonist to probe GPR109A function, off-target effects cannot be fully excluded. Further studies using genetic knockout models may help validate these findings.

From a translational perspective, as a metabolic sensor for microbiota- and diet-derived short-chain fatty acids, particularly butyrate, GPR109A can be modulated through dietary interventions that enhance fermentable fiber intake and endogenous butyrate production. In parallel, microbiota-based approaches, including butyrate-producing probiotics or postbiotic formulations, may reinforce GPR109A-mediated epithelial protection, especially in individuals with impaired microbial fermentation capacity. Moreover, pharmacological activation of GPR109A could be explored as an adjunctive strategy in high-risk periodontitis patients with systemic inflammatory or metabolic comorbidities, thereby translating mechanistic insights into clinically actionable interventions. Additionally, monitoring GPR109A expression or fecal butyrate levels might offer a potential approach to assess gut barrier risks in periodontitis patients. Nevertheless, the clinical relevance of targeting GPR109A will require further validation in well-characterized human cohorts to establish its therapeutic potential.

In conclusion, this study demonstrates that periodontitis promotes intestinal inflammation through gut microbiota–mediated suppression of colonic GPR109A. We show that downregulation of this receptor disrupts epithelial barrier integrity and enhances pro-inflammatory responses, whereas its activation restores barrier function and attenuates inflammation. These findings not only advance our mechanistic understanding of the oral–gut axis but also highlight GPR109A as a promising therapeutic target for mitigating the intestinal consequences of periodontitis.

## Data Availability

The raw data supporting the conclusions of this article will be made available by the authors, without undue reservation.
