# Supplementary material for: Periodontitis promotes intestinal inflammation through gut microbiota–mediated suppression of GPR109A
Source: Front Cell Infect Microbiol. 2026 Feb 24;16:1761932. doi: 10.3389/fcimb.2026.1761932 (PMC12971913; doi:10.3389/fcimb.2026.1761932)
Supplement: Supplementary file 1 [file Table1.docx]

Supplementary Material

Table S1. Prime sequences used in RT-PCR.

| Primer name | Forward primer sequence (5’-3’) | Reverse primer sequence (5’-3’) |  |
| --- | --- | --- | --- |
| GPR109A | CTGTTTCCACCTCAAGTCCTGG | CATAGTTGTCCGTCAGGAACGG |  |
| TNF-α | AACTCCAGGCGGTGCCTAT | TGCCACAAGCAGGAATGAGA |  |
| IL-1β | TCGCTCAGGGTCACAAGAAA | CATCAGAGGCAAGGAGGAAAAC |  |
| IL-2 | CCTGAGCAGGATGGAGAATTACA | TCCAGAACATGCCGCAGAG |  |
| IL-6 | AGTTGCCTTCTTGGGACTGA | TCCACGATTTCCCAGAGAAC | |
| IL-17A | TGACGCCCACCTACAACATC | CATCATGCAGTTCCGTCAGC | |
| IL-23 | ATGCTGGATTGCAGAGCAGTA | ACGGGGCACATTATTTTTAGTCT |  |
| IFN-γ | TGGCTGTTTCTGGCTTGTTACT | TGACGCTTATGTTGTTGCTGA |  |
| Csf1 | GTGTCAGAACACTGTAGCCAC | TCAAAGGCAATCTGGCATGAAG | |
| Cxcl1 | GGCGCCTATCGCCAATG | CTGGATGTTCTTGAGGTGAATCC | |
| ZO-1 | ACAGGCCATTACGAGCCTCT | GGAGGCTGTGGTTTGGTAGC |  |
| Occludin | ATTCCATCAGTTTCCTATCT | ACCAGGACCTTTCTTGAC |  |
| Jam3 | CACTACAGCTGGTACCGCAATG | CTGGGATTGGCTCTGGAATC |  |
| Cldn2 | TCTCAGCCCTGTTTTCTTTGG | GGCGAGCAGGAAAAGCAA |  |
| Cldn3 | TCATCACGGCGCAGATCA | CTCTGCACCACGCAGTTCA |  |
| GAPDH | AACGACCCCTTCATTGAC | TCCACGACATACTCAGCAC |  |


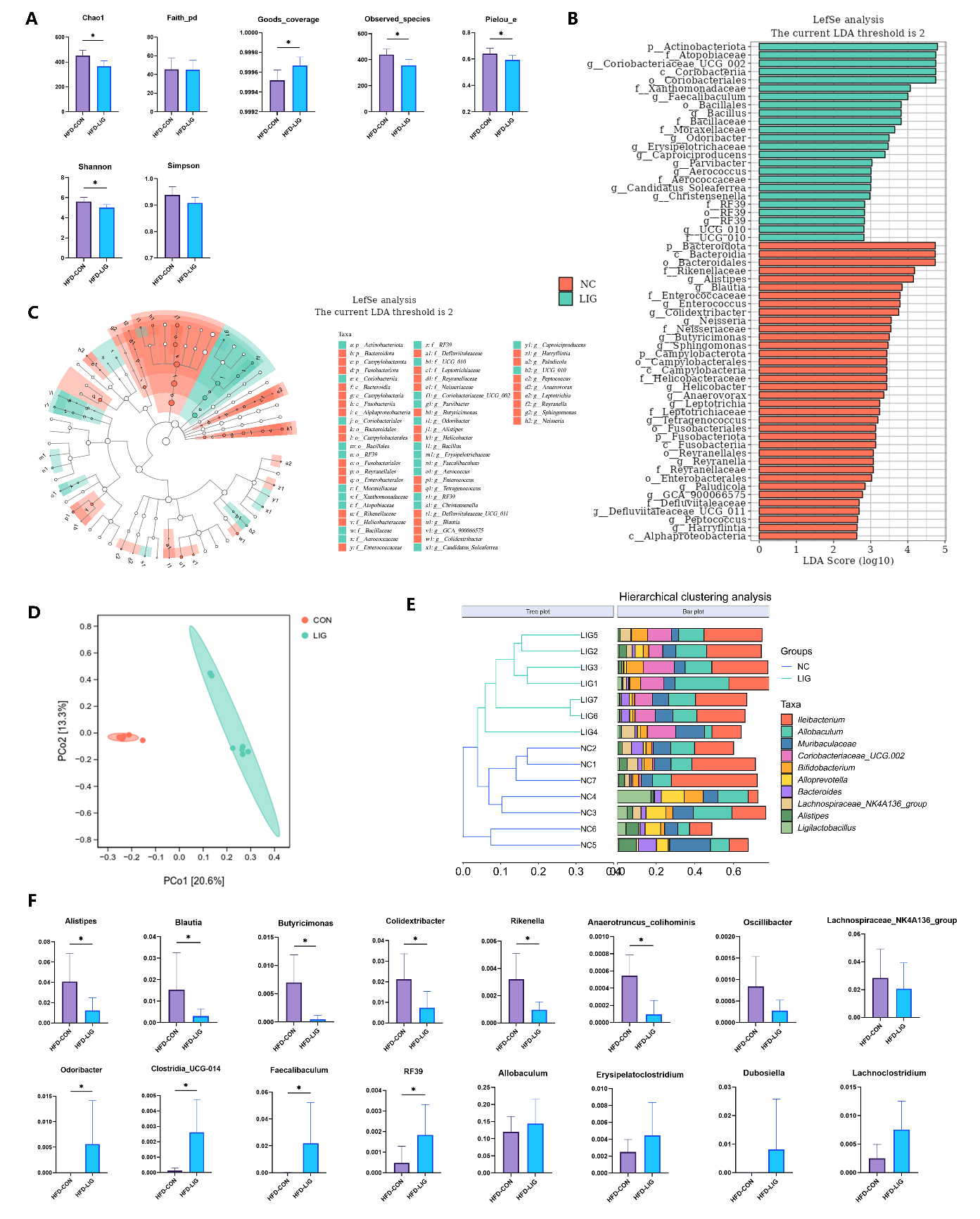


**Supplementary Figure 1. Periodontitis induces gut microbiota dysbiosis in HFD-fed donor mice.** (A) Alpha diversity analysis shows a significant decrease in bacterial diversity in the HFD_LIG group compared with the HFD_CON group. (B, C) Linear discriminant analysis Effect Size (LEfSe) analysis identifies differentially abundant bacterial taxa between the two groups (LDA score > 2.0). (D, E) Beta diversity analysis is visualized by Principal Coordinate Analysis (PCoA) and hierarchical clustering analysis based on distance matrices, which reveal distinct clustering and significant separation of microbial community structures between the two groups. (F) Comparative analysis of SCFA-producing bacteria demonstrates statistical differences in the relative abundance of key genera involved in short-chain fatty acid production. Data are presented as mean ± SD (n = 7). *P < 0.05. HFD_CON, high-fructose diet control; HFD_LIG, high-fructose diet and periodontitis.
